# Supplementary material for: APOE genotype influences on the brain metabolome of aging mice – role for mitochondrial energetics in mechanisms of resilience in APOE2 genotype
Source: Mol Neurodegener. 2025 Sep 2;20:97. doi: 10.1186/s13024-025-00888-z (PMC12403941; doi:10.1186/s13024-025-00888-z)
Supplement: Supplementary file 1 — Supplementary Material 1 [file 13024_2025_888_MOESM1_ESM.pdf]

Table S1. The demographics of the ROS-MAP cohort, subset used for the analysis.

	ApoE2/3	ApoE3/3	ApoE 3/4
n=	66	304	112
Age at death (mean ± std eva)	92.3±6.6	90.5±6.35	89.5±5.5
BMI (lastest data available)	24.1±5.08	26.2±5.28	25.4±4.54
Education	15.4±3.11	15.9±3.41	16.3±3.24
PMI	7.51±4.01	8.5±5.36	7.29±3.91
Sex (Male %)	18.18 %	31.25 %	32.14 %
Final consensus cognitive diagnosis			
NCI: No cognitive impairment (No impaired domains)	41%	32%	18%
MCI: Mild cognitive impairment (One impaired domain) and NO other cause of CI	17%	27%	19%
MCI: Mild cognitive impairment (One impaired domain) AND another cause of CI	5%	0%	1%
AD: Alzheimer's dementia and NO other cause of CI (NINCDS PROB AD)	30%	35%	52%
AD: Alzheimer's dementia AND another cause of CI (NINCDS POSS AD)	8%	5%	8%
Other dementia: Other primary cause of dementia	0%	2%	3%
