# Supplementary material for: APOE genotype influences on the brain metabolome of aging mice – role for mitochondrial energetics in mechanisms of resilience in APOE2 genotype
Source: Mol Neurodegener. 2025 Sep 2;20:97. doi: 10.1186/s13024-025-00888-z (PMC12403941; doi:10.1186/s13024-025-00888-z)
Supplement: Supplementary file 2 — Supplementary Material 2 [file 13024_2025_888_MOESM2_ESM.pdf]

Table S4. Cluster components of selected ROS-MAP metabolites.

Compound	Cluster information				P values - Full factorial model on cluster components							
	**Cluster**	**RSquare with Own Cluster**	**RSquare with Next Closest**	**1-RSquare Ratio**	**Age at death**	**BMI**	**APOE Genotypes**	**Beta-hydroxyisovaleroylcarnitine**	**Cognition diagnosis**	**Education**	**Sex**	**PMI**
(S)-3-hydroxybutyrylcarnitine	1	0.626	0.072	0.402	0.0934	0.9953	**0.0002**	**<.0001**	0.9502	0.4869	0.2048	**<.0001**
acetylcarnitine (C2)		0.635	0.106	0.409								
arachidonoylcarnitine (C20:4)		0.389	0.001	0.611								
carnitine		0.487	0.113	0.578								
eicosenoylcarnitine (C20:1)*		0.616	0.142	0.448								
tiglyl carnitine (C5:1)		0.552	0.266	0.61								
3-methylglutaconate	2	0.668	0.116	0.375	0.5889	0.2374	**0.0023**	**<.0001**	0.5082	0.1103	0.0573	**0.0100**
alpha-hydroxyisovalerate		0.612	0.198	0.484								
methylmalonate (MMA)		0.396	0.019	0.615								
