# Supplementary material for: APOE genotype influences on the brain metabolome of aging mice – role for mitochondrial energetics in mechanisms of resilience in APOE2 genotype
Source: Mol Neurodegener. 2025 Sep 2;20:97. doi: 10.1186/s13024-025-00888-z (PMC12403941; doi:10.1186/s13024-025-00888-z)
Supplement: Supplementary file 3 — Supplementary Material 3 [file 13024_2025_888_MOESM3_ESM.pdf]

Table S3. APOE genotypes influenced multiple metabolism pathways in the brains in the ROS-MAP cohort. ANCOVA analysis was performed on medication-adjusted metabolomic data, controlling BMI, PMI, age at death, education, sex, cognitive diagnosis, and beta-hydroxyisovaleroylcarnitine, followed by Tukey pos-hoc test, where the levels were not decorated with the same letters were significantly different from each other (i.e., A and B).

Metabolite	APOE genotype			p-value	SUPER_PATHWAY	SUB_PATHWAY
	e2e3	e3e3	e3e4			
carnitine	B	A	A	0.0042	Lipid	Carnitine Metabolism
(S)-3-hydroxybutyrylcarnitine	B	A	A	0.0075	Lipid	Fatty Acid Metabolism (Acyl Carnitine, Hydroxy)
eicosenoylcarnitine (C20:1)*	B	AB	A	0.0412	Lipid	Fatty Acid Metabolism (Acyl Carnitine, Monounsaturated)
arachidonoylcarnitine (C20:4)	B	AB	A	0.0254	Lipid	Fatty Acid Metabolism (Acyl Carnitine, Polyunsaturated)
acetylcarnitine (C2)	B	A	A	0.0033	Lipid	Fatty Acid Metabolism (Acyl Carnitine, Short Chain)
methylmalonate (MMA)	AB	A	B	0.0049	Lipid	Fatty Acid Metabolism (also BCAA Metabolism)
alpha-hydroxyisovalerate	B	A	AB	0.0215	Amino Acid	Leucine, Isoleucine and Valine Metabolism
tiglyl carnitine (C5)	B	A	AB	0.0265	Amino Acid	Leucine, Isoleucine and Valine Metabolism
3-methylglutaconate	B	A	AB	0.0471	Lipid	Leucine, Isoleucine and Valine Metabolism
N-behenoyl-sphingadienine (d18:2/22:0)*	A	A	A	0.0330	Lipid	Ceramides
stearoyl-docosahexaenoyl-glycerol (18:0/22:6) [1]*	A	B	AB	0.0348	Lipid	Diacylglycerol
1-stearoyl-GPC (18:0)	A	B	AB	0.0038	Lipid	Lysophospholipid
1-palmitoyl-GPC (16:0)	A	A	A	0.0490	Lipid	Lysophospholipid
1-oleoyl-2-docosahexaenoyl-GPC (18:1/22:6)*	A	A	A	0.0290	Amino Acid	Phosphatidylcholine (PC)
1-palmitoyl-2-docosahexaenoyl-GPC (16:0/22:6)	AB	B	A	0.0230	Lipid	Phosphatidylcholine (PC)
N-acetylglucosamine/N-acetylgalactosamine	A	AB	B	0.0480	Carbohydrate	Aminosugar Metabolism
benzoate	A	AB	B	0.0485	Xenobiotics	Benzoate Metabolism
arachidonoyl ethanolamide	A	B	B	0.0142	Lipid	Endocannabinoid
methyl glucopyranoside (alpha + beta)	A	A	B	0.0022	Xenobiotics	Food Component/Plant
3-hydroxystachydrine*	A	AB	B	0.0071	Xenobiotics	Food Component/Plant
stachydrine	A	B	B	0.0087	Xenobiotics	Food Component/Plant
ophthalmate	A	A	A	0.0498	Amino Acid	Glutathione Metabolism
imidazole propionate	AB	B	A	0.0231	Amino Acid	Histidine Metabolism
chiro-inositol	A	AB	B	0.0368	Lipid	Inositol Metabolism
S-adenosylhomocysteine (SAH)	B	A	AB	0.0350	Amino Acid	Methionine, Cysteine, SAM and Taurine Metabolism
2,3-dihydroxy-5-methylthio-4-pentenoate (DMTPA)*	B	A	A	0.0095	Lipid	Methionine, Cysteine, SAM and Taurine Metabolism
2'-deoxycytidine	A	AB	B	0.0355	Amino Acid	Pyrimidine Metabolism, Cytidine containing
cytidine	A	AB	B	0.0440	Nucleotide	Pyrimidine Metabolism, Cytidine containing
kynurenate	B	A	A	0.0228	Amino Acid	Tryptophan Metabolism
kynurenine	B	A	A	0.0232	Amino Acid	Tryptophan Metabolism
tyrosine	B	A	AB	0.0408	Amino Acid	Tyrosine Metabolism
pyridoxal	A	B	AB	0.0431	Cofactors and Vitamins	Vitamin B6 Metabolism
X - 24431	A	B	AB	0.0078	0	0
X - 23171	AB	B	A	0.0312	0	0
X - 24807	A	B	AB	0.0427	0	0
X - 12104	A	A	A	0.0472	0	0
