# Supplementary figures and images for: APOE genotype influences on the brain metabolome of aging mice – role for mitochondrial energetics in mechanisms of resilience in APOE2 genotype
Source: Mol Neurodegener. 2025 Sep 2;20:97. doi: 10.1186/s13024-025-00888-z (PMC12403941; doi:10.1186/s13024-025-00888-z)

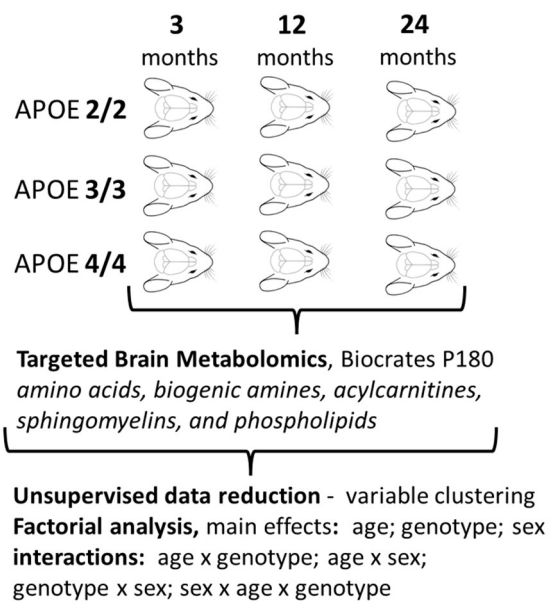

**Figure S1. Experimental design.**

Supplement: Supplementary file 4 — Supplementary Material 4 [file 13024_2025_888_MOESM4_ESM.pdf]
