# Supplementary material for: APOE genotype influences on the brain metabolome of aging mice – role for mitochondrial energetics in mechanisms of resilience in APOE2 genotype
Source: Mol Neurodegener. 2025 Sep 2;20:97. doi: 10.1186/s13024-025-00888-z (PMC12403941; doi:10.1186/s13024-025-00888-z)
Supplement: Supplementary file 5 — Supplementary Material 5 [file 13024_2025_888_MOESM5_ESM.pdf]

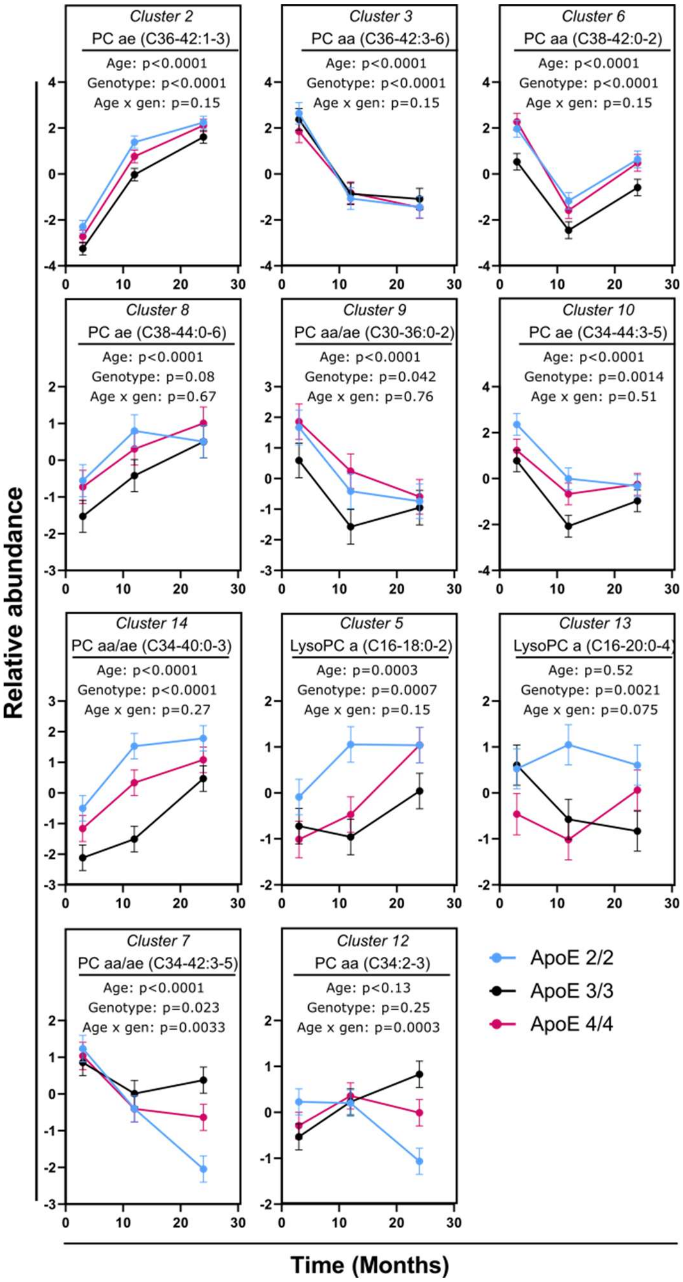

Figure S2. Aging pattern of phospholipids between APOE genotypes. To reveal the intercorrelation structure and to facilitate interpretation, data were reduced using unsupervised variable clustering and converted into cluster components. Supplementary Table S2 contains a detailed cluster description, including the correlation between metabolites within each cluster.
