# Supplementary material for: APOE genotype influences on the brain metabolome of aging mice – role for mitochondrial energetics in mechanisms of resilience in APOE2 genotype
Source: Mol Neurodegener. 2025 Sep 2;20:97. doi: 10.1186/s13024-025-00888-z (PMC12403941; doi:10.1186/s13024-025-00888-z)
Supplement: Supplementary file 6 — Supplementary Material 6 [file 13024_2025_888_MOESM6_ESM.pdf]

Supplemental Table S2. Describtion of cluster analysis with each cluster membership. P values from factorial analysis performed on individual metabolites and cluster components.

Class	Compound	Cluster information				P values - Full factorial model on individual metabolites							P values - Full factorial model on cluster components			
		Cluster	RSquare with Own Cluster	RSquare with Next Closest	1-RSquare Ratio	Age	Genotype	Gender	Age*Gender	Genotype*Age	Genotype*Age *Gender	Genotype*Gen der	Age	Gender	Genotype	Genotype*Age
Amino Acids	Asn		0.73	0.40	0.46	0.0001	0.53	0.17	0.74	0.0043	0.65	0.71				
	Glu		0.70	0.32	0.44	0.0001	0.22	0.39	0.87	0.92	0.3	0.13				
	Asp	**1**	0.44	0.11	0.63	0.0029	0.042	0.2	0.034	0.012	0.88	0.67	0.0001	0.11	0.55	0.15
	Thr		0.41	0.22	0.75	0.0001	0.07	0.0019	0.56	0.092	0.86	0.57				
	Ala		0.35	0.27	0.90	0.45	0.13	0.52	0.37	0.021	0.71	0.98				
	Phe		0.86	0.53	0.29	0.6	0.0001	0.053	0.53	0.0005	0.54	0.35				
	His	**2**	0.83	0.27	0.23	0.82	0.046	0.22	0.27	0.02	0.16	0.067	0.047	0.83	0.0018	0.034
	Gln		0.79	0.18	0.25	0.0001	0.098	0.92	0.38	0.6	0.48	0.049				
	Trp		0.64	0.18	0.44	0.0001	0.0027	0.0053	0.6	0.032	0.66	0.29				
	Met		0.79	0.28	0.29	0.0001	0.0001	0.25	0.11	0.5	0.096	0.3				
	Tyr	**3**	0.76	0.22	0.31	0.0001	0.16	0.0001	0.058	0.11	0.1	0.26	0.0001	0.0001	0.048	0.014
	Pro		0.69	0.47	0.60	0.0001	0.025	0.0035	0.59	0.0001	0.24	0.46				
	t4-OH-Pro		0.37	0.18	0.77	0.0001	0.0004	0.0001	0.21	0.0063	0.53	0.47				
	Gly		0.78	0.39	0.36	0.0001	0.0001	0.2	0.88	0.0007	0.57	0.016				
	Lys	**4**	0.75	0.52	0.51	0.0001	0.0001	0.95	0.71	0.023	0.0087	0.0074	0.0001	0.041	0.0003	0.092
	Ser		0.70	0.27	0.41	0.049	0.0001	0.0001	0.94	0.14	0.44	0.096				
	alpha-AAA		0.47	0.28	0.74	0.0001	0.017	0.39	0.92	0.67	0.14	0.011				
	Val		0.91	0.39	0.15	0.35	0.0001	0.0001	0.55	0.0009	0.2	0.027				
	Ile	**5**	0.83	0.28	0.23	0.11	0.0001	0.011	0.63	0.0001	0.54	0.41	0.78	0.0001	0.0001	0.0002
	Arg		0.67	0.46	0.62	0.074	0.0001	0.0012	0.75	0.0006	0.16	0.016				
Biogenic Amines	Spermidine		0.93	0.07	0.07	0.0011	0.012	0.88	0.69	0.092	0.12	0.97				
	Spermine	**1**	0.88	0.01	0.12	0.25	0.064	0.77	0.27	0.2	0.12	0.83	0.0012	0.95	0.001	0.082
	Putrescine		0.68	0.10	0.36	0.0001	0.0001	0.7	0.024	0.077	0.0007	0.81				
	Taurine		0.77	0.01	0.23	0.0002	0.13	0.0001	0.42	0.91	0.078	0.32				
	Creatinine	**2**	0.72	0.01	0.29	0.029	0.067	0.012	0.62	0.032	0.15	0.75	0.0001	0.0001	0.049	0.23
	Serotonin		0.49	0.00	0.51	0.0001	0.069	0.61	0.12	0.22	0.26	0.32				
	Carnosine	**3**	0.79	0.04	0.22	0.0001	0.35	0.0005	0.072	0.69	0.12	0.38	0.0001	0.0001	0.55	0.21
	SDMA		0.79	0.05	0.22	0.0001	0.94	0.0061	0.97	0.057	0.0018	0.39				
	C10:2		0.85	0.47	0.28	0.0046	0.36	0.2	0.76	0.49	0.33	0.52				
	C14:2		0.85	0.70	0.51	0.45	0.22	0.25	0.36	0.39	0.069	0.71				
	C14:2-OH		0.89	0.69	0.35	0.34	0.14	0.2	0.55	0.57	0.11	0.7				
	C16:2		0.83	0.79	0.80	0.72	0.52	0.14	0.34	0.54	0.36	0.66				
	C3:1		0.92	0.42	0.14	0.0005	0.13	0.6	1	0.6	0.075	0.54				
	C3-OH		0.92	0.44	0.15	0.0086	0.068	0.4	0.99	0.57	0.1	0.51				
	C4:1		0.90	0.44	0.19	0.016	0.15	0.83	0.97	0.45	0.13	0.46				
	C5:1		0.94	0.46	0.12	0.0013	0.096	0.47	0.99	0.55	0.24	0.52				
	C5:1-DC		0.94	0.45	0.11	0.0003	0.092	0.53	0.96	0.7	0.11	0.41				
	C5-DC (C6-OH)	**1**	0.80	0.55	0.45	0.071	0.2	0.61	0.69	0.18	0.3	0.46	0.016	0.32	0.2	0.54
	C5-M-DC		0.90	0.44	0.18	0.0027	0.039	0.61	0.98	0.37	0.1	0.42				
	C6 (C4:1-DC)		0.84	0.56	0.36	0.19	0.51	0.1	0.84	0.78	0.19	0.32				
	C6:1		0.94	0.46	0.10	0.0027	0.34	0.35	0.96	0.32	0.27	0.53				
	C7-DC		0.89	0.57	0.26	0.072	0.22	0.07	0.57	0.13	0.25	0.63				
	C9		0.91	0.53	0.20	0.0034	0.28	0.13	0.33	0.54	0.13	0.53				
	Sum of Medium-Chain ACs		0.88	0.54	0.26	0.011	0.31	0.15	0.74	0.82	0.15	0.33				
	C12		0.85	0.53	0.32	0.0001	0.0004	0.0076	0.019	0.61	0.36	0.48				
	C14		0.92	0.43	0.14	0.0001	0.0001	0.15	0.079	0.12	0.43	0.17				
	C16	**2**	0.94	0.51	0.12	0.0001	0.0001	0.24	0.16	0.062	0.65	0.38	0.0001	0.69	0.0001	0.48
	C16:1		0.93	0.55	0.16	0.0001	0.0001	0.054	0.0096	0.69	0.42	0.16				
	C18		0.62	0.35	0.58	0.0001	0.077	0.01	0.9	0.0011	0.33	0.67				
	C18:1		0.97	0.42	0.06	0.0001	0.0001	0.64	0.085	0.29	0.74	0.17				

**Acyl carnitines**	C14:1		0.78	0.62	0.58	0.0001	0.024	0.043	0.021	0.75	0.21	0.47				
	C14:1-OH		0.92	0.68	0.26	0.11	0.015	0.074	0.29	0.33	0.29	0.34				
	C16:1-OH		0.91	0.56	0.21	0.0003	0.0027	0.28	0.31	0.47	0.31	0.15				
	C16:2-OH		0.87	0.70	0.42	0.1	0.023	0.18	0.29	0.41	0.18	0.55				
	C16-OH		0.83	0.53	0.36	0.074	0.086	0.16	0.048	0.046	0.11	0.85				
	C18:1-OH		0.83	0.58	0.41	0.57	0.4	0.15	0.018	0.73	0.14	0.57				
	C18:2	**3**	0.63	0.56	0.85	0.0001	0.0049	0.29	0.22	0.23	0.74	0.36	0.022	0.12	0.039	0.54
	Sum of ACs		0.91	0.65	0.25	0.41	0.012	0.11	0.16	0.85	0.35	0.14				
	Sum of Long-Chain ACs		0.91	0.63	0.25	0.0001	0.0081	0.16	0.15	0.32	0.6	0.26				
	Sum of MUFA-ACs		0.92	0.68	0.26	0.42	0.031	0.21	0.24	0.45	0.29	0.26				
	Sum of PUFA-ACs		0.86	0.57	0.33	0.37	0.095	0.12	0.41	0.54	0.43	0.35				
	2MBG (NBS)		0.75	0.21	0.32	0.0001	0.0001	0.26	0.82	0.0001	0.33	0.33				
	C5		0.67	0.35	0.51	0.0001	0.0001	0.86	0.27	0.0001	0.6	0.039				
	IVA (NBS)		0.81	0.21	0.24	0.0001	0.0001	0.17	0.35	0.0001	0.52	0.97				
	SBCAD Deficiency (NBS)	**4**	0.79	0.36	0.32	0.0001	0.0001	0.076	0.33	0.0001	0.86	0.32	0.0001	0.17	0.0001	0.0001
	SCAD Deficiency (NBS)		0.24	0.07	0.82	0.0001	0.63	0.85	0.3	0.16	0.075	0.28				
	3MGA (NBS)		0.65	0.09	0.38	0.0067	0.22	0.18	0.44	0.9	0.4	0.36				
	BKT Deficiency (NBS)		0.57	0.11	0.48	0.0004	0.001	0.41	0.21	0.019	0.15	0.24				
	b-Oxidation	**5**	0.70	0.33	0.45	0.0004	0.21	0.12	0.44	0.062	0.84	0.14	0.0001	0.022	0.032	0.41
	CPT-1 Deficiency (NBS)		0.44	0.39	0.91	0.0001	0.54	0.0001	0.0011	0.0001	0.12	0.022				
	Ratio of Acetylcarnitine to Carnitine		0.65	0.06	0.38	0.0008	0.0099	0.082	0.69	0.0008	0.13	0.1				
	IBD Deficiency (NBS)	**6**	0.78	0.19	0.27	0.0001	0.031	0.076	0.057	0.0001	0.14	0.95	0.0007	0.15	0.013	0.0014
	MA (NBS)		0.89	0.17	0.13	0.085	0.0016	0.27	0.021	0.0051	0.0078	0.57				
	MMA (NBS)		0.73	0.35	0.42	0.0022	0.098	0.3	0.14	0.05	0.13	0.41				
	C0		0.74	0.38	0.43	0.0058	0.0009	0.013	0.054	0.0019	0.99	0.089				
	C2		0.67	0.23	0.43	0.68	0.0001	0.075	0.0027	0.0001	0.37	0.16				
	C3-DC (C4-OH)		0.57	0.31	0.62	0.047	0.03	0.0027	0.0001	0.0001	0.45	0.36				
	C5-OH (C3-DC-M)	**7**	0.79	0.49	0.41	0.086	0.007	0.14	0.085	0.044	0.41	0.042	0.58	0.021	0.0008	0.003
	Sum of Short-Chain Acs		0.79	0.66	0.63	0.14	0.0079	0.11	0.15	0.52	0.31	0.051				
	CACT Deficiency (NBS)		0.59	0.40	0.69	0.0001	0.0049	0.0053	0.71	0.0001	0.98	0.67				
	LCHAD Deficiency (NBS)		0.74	0.32	0.39	0.0001	0.089	0.048	0.8	0.052	0.0019	0.097				
	Ratio of Medium-Chain to Long-Chain ACs	**8**	0.82	0.49	0.36	0.0001	0.022	0.087	0.074	0.17	0.62	0.97	0.0001	0.0029	0.36	0.024
	VLCAD Deficiency (NBS)		0.65	0.36	0.54	0.0001	0.16	0.0006	0.34	0.014	0.063	0.25				
	w-Oxidation		0.53	0.30	0.67	0.0001	0.089	0.6	0.69	0.0096	0.31	0.76				

	Ratio of Short-Chain to Medium-Chain ACs	9	0.85	0.22	0.19	0.0029	0.14	0.5	0.92	0.093	0.64	0.63	0.0001	0.089	0.06	0.15
	TFP Deficiency (NBS)		0.85	0.45	0.27	0.0001	0.051	0.0097	0.046	0.23	0.53	0.87				
	CPT-2 Deficiency (NBS)	10	0.58	0.27	0.57	0.0001	0.12	0.001	0.097	0.0001	0.13	0.51	0.0001	0.0073	0.33	0.0001
	MC Deficiency (NBS)		0.60	0.27	0.56	0.0001	0.03	0.089	0.41	0.0009	0.28	0.084				
	PA (NBS)		0.66	0.29	0.47	0.0001	0.063	0.029	0.054	0.0013	0.28	0.23				
	Ratio of Short-Chain to Long-Chain ACs		0.67	0.43	0.58	0.0001	0.62	0.81	0.88	0.0033	0.61	0.66				
	C3	11	0.91	0.23	0.11	0.21	0.61	0.2	0.84	0.012	0.58	0.048	0.0028	0.26	0.41	0.0039
	C4		0.91	0.30	0.12	0.0001	0.3	0.36	0.36	0.0024	0.4	0.05				
osphatidylcholines	PC aa C36:4	1	0.98	0.60	0.05	0.011	0.61	0.57	0.53	0.68	0.34	0.85	0.12	0.47	0.56	0.8
	PC aa C32:0		0.99	0.61	0.02	0.15	0.41	0.44	0.38	0.74	0.36	0.91				
	PC aa C38:4		0.98	0.61	0.05	0.02	0.6	0.67	0.39	0.75	0.36	0.89				
	PC aa C32:1		0.92	0.53	0.18	0.091	0.94	0.19	0.44	0.76	0.14	0.73				
	PC aa C36:1		0.99	0.57	0.02	0.15	0.52	0.46	0.4	0.77	0.39	0.86				
	PC aa C38:6		0.98	0.60	0.04	0.16	0.62	0.25	0.28	0.77	0.32	0.98				
	PC aa C40:6		0.92	0.59	0.20	0.016	0.82	0.37	0.22	0.79	0.33	0.97				
	PC aa C34:1		0.97	0.55	0.07	0.16	0.48	0.98	0.38	0.84	0.25	0.77				
	PC aa C36:2		0.90	0.46	0.19	0.28	0.048	0.59	0.2	0.91	0.38	0.81				
	PC ae C42:2	2	0.84	0.60	0.39	0.0001	0.0008	0.0001	0.0041	0.036	0.047	0.14	0.0001	0.0001	0.0001	0.71
	PC ae C36:3		0.93	0.49	0.15	0.0001	0.2	0.0001	0.29	0.13	0.78	0.15				
	PC ae C38:3		0.95	0.58	0.13	0.0001	0.011	0.0001	0.093	0.18	0.97	0.14				
	PC ae C38:1		0.92	0.74	0.30	0.0001	0.0001	0.019	0.02	0.58	0.56	0.13				
	PC ae C38:2		0.92	0.77	0.33	0.0001	0.0001	0.12	0.43	0.64	0.79	0.41				
	PC ae C40:2		0.94	0.64	0.15	0.0001	0.0001	0.0001	0.0001	0.66	0.22	0.08				
	PC aa C42:6	3	0.82	0.41	0.31	0.0001	0.042	0.88	0.65	0.13	0.14	1	0.0001	0.19	0.76	0.84
	PC aa C38:3		0.82	0.58	0.43	0.0001	0.057	0.79	0.67	0.2	0.72	0.22				
	PC aa C40:4		0.85	0.41	0.25	0.0001	0.34	0.0027	0.98	0.34	0.35	0.79				
	PC aa C42:4		0.79	0.56	0.49	0.0001	0.81	0.79	0.23	0.49	0.098	0.89				
	PC aa C42:5		0.81	0.54	0.40	0.0001	0.33	0.95	0.24	0.79	0.12	0.14				
	PC aa C38:5		0.71	0.57	0.67	0.0001	0.84	0.97	0.78	0.79	0.28	0.3				
	PC aa C40:5		0.54	0.33	0.70	0.0001	0.035	0.64	0.77	0.85	0.32	0.91				
	PC ae C36:5		0.72	0.40	0.47	0.0001	0.71	0.0001	0.76	0.91	0.83	0.42				
	PC aa C26:0	4	0.88	0.44	0.22	0.077	0.43	0.93	0.11	0.067	0.94	0.97	0.96	0.55	0.38	0.27
	PC aa C24:0		0.85	0.59	0.36	0.092	0.61	0.62	0.22	0.088	0.92	0.94				
	PC aa C28:1		0.78	0.57	0.51	0.0004	0.02	0.3	0.78	0.11	0.6	0.69				
	lysoPC a C24:0		0.77	0.51	0.46	0.0088	0.45	0.27	0.81	0.54	0.83	0.81				
	lysoPC a C26:0		0.89	0.38	0.18	0.99	0.83	0.88	0.085	0.55	0.97	0.74				
	lysoPC a C28:0		0.88	0.40	0.19	0.83	0.39	0.11	0.12	0.67	0.98	0.92				
	lysoPC a C18:2	5	0.60	0.37	0.64	0.036	0.12	0.084	0.29	0.03	0.21	0.38	0.0003	0.074	0.0007	0.15
	lysoPC a C18:0		0.76	0.36	0.37	0.0001	0.0001	0.38	0.93	0.13	0.75	0.046				
	lysoPC a C16:1		0.80	0.69	0.65	0.0007	0.02	0.0013	0.21	0.19	0.18	0.4				
	lysoPC a C17:0		0.80	0.38	0.33	0.081	0.0003	0.054	0.56	0.33	0.36	0.12				
	PC aa C42:0	6	0.48	0.18	0.64	0.0001	0.11	0.34	0.46	0.18	0.065	0.61	0.0001	0.0001	0.0001	0.79
	PC ae C42:1		0.87	0.23	0.16	0.0001	0.0001	0.0001	0.027	0.29	0.0018	0.63				
	PC aa C42:2		0.76	0.35	0.37	0.0001	0.0002	0.0001	0.19	0.38	0.11	0.33				
	PC aa C42:1		0.80	0.25	0.26	0.0001	0.0087	0.0001	0.52	0.4	0.025	0.29				
	PC aa C40:1		0.93	0.25	0.10	0.0001	0.0001	0.0001	0.05	0.67	0.046	0.34				
	PC aa C38:0		0.71	0.38	0.47	0.0001	0.0001	0.031	0.028	0.67	0.46	0.64				
	PC aa C36:5	7	0.83	0.25	0.23	0.0001	0.0001	0.0001	0.2	0.0001	0.22	0.14	0.0001	0.0001	0.022	0.0033
	PC aa C36:3		0.77	0.47	0.44	0.0001	0.0072	0.0005	0.53	0.0008	0.67	0.37				
	PC aa C34:4		0.78	0.38	0.36	0.14	0.16	0.0004	0.23	0.026	0.13	0.048				
	PC ae C42:3		0.76	0.40	0.40	0.0001	0.72	0.0045	0.31	0.28	0.56	0.31				
	PC aa C40:3		0.60	0.46	0.75	0.001	0.11	0.0001	0.0001	0.0002	0.66	0.062				
	PC ae C44:4		0.50	0.27	0.68	0.0001	0.12	0.0001	0.019	0.069	0.1	0.57				

Pl	PC ae C38:0	8	0.66	0.36	0.53	0.0001	0.25	0.0001	0.052	0.1	0.39	0.89	0.0001	0.0001	0.08	0.67
	PC ae C40:1		0.67	0.51	0.68	0.0001	0.0001	0.0001	0.1	0.52	0.52	0.79				
	PC ae C40:6		0.52	0.33	0.72	0.1	0.037	0.0005	0.28	0.54	0.55	0.4				
	PC ae C44:6		0.41	0.28	0.82	0.94	0.2	0.0001	0.011	0.56	0.0053	0.72				
	PC ae C44:5		0.73	0.40	0.45	0.0001	0.85	0.0002	0.18	0.68	0.75	0.55				
	PC ae C42:0		0.42	0.25	0.76	0.57	0.024	0.82	0.54	0.72	0.35	0.95				
	PC aa C36:6	9	0.43	0.27	0.77	0.0033	0.0089	0.0001	0.32	0.013	0.044	0.92	0.0001	0.12	0.042	0.76
	PC ae C30:1		0.81	0.39	0.31	0.0001	0.022	0.11	0.65	0.37	0.33	0.96				
	PC ae C30:2		0.78	0.50	0.44	0.0003	0.13	0.12	0.54	0.65	0.12	0.76				
	PC ae C36:0		0.79	0.47	0.39	0.0008	0.0036	0.34	0.14	0.66	0.29	0.96				
	PC aa C30:0		0.84	0.61	0.42	0.044	0.066	0.048	0.14	0.74	0.023	0.66				
	PC ae C32:1		0.73	0.47	0.51	0.0001	0.25	0.16	0.85	0.78	0.09	0.85				
	PC ae C30:0		0.92	0.61	0.21	0.001	0.02	0.15	0.32	0.83	0.023	0.47				
	PC ae C34:0		0.80	0.39	0.32	0.0001	0.037	0.24	0.24	0.88	0.2	0.83				
	PC ae C40:5	10	0.64	0.41	0.61	0.0001	0.21	0.033	0.25	0.0001	0.63	0.029	0.0001	0.67	0.0014	0.51
	PC ae C40:4		0.72	0.36	0.44	0.014	0.0008	0.47	0.63	0.084	0.63	0.053				
	PC ae C34:1		0.78	0.29	0.30	0.0001	0.0001	0.64	0.66	0.099	0.61	0.0011				
	PC ae C38:4		0.81	0.35	0.29	0.0001	0.047	0.61	0.51	0.27	0.42	0.004				
	PC ae C44:3		0.50	0.42	0.86	0.0001	0.0088	0.28	0.13	0.44	0.15	0.23				
	PC ae C36:4		0.74	0.50	0.53	0.0001	0.033	0.001	0.02	0.67	0.99	0.011				
	PC ae C38:5		0.67	0.26	0.45	0.0001	0.011	0.034	0.25	0.88	0.92	0.024				
	PC ae C34:3	11	0.68	0.29	0.45	0.0018	0.94	0.12	0.95	0.0068	0.1	0.84	0.98	0.39	0.6	0.2
	PC ae C32:2		0.60	0.34	0.60	0.0013	0.012	0.14	0.58	0.072	0.65	0.16				
	lysoPC a C28:1		0.72	0.42	0.49	0.0001	0.46	0.93	0.36	0.39	0.12	0.9				
	PC aa C32:3		0.76	0.52	0.50	0.3	0.8	0.45	0.55	0.47	0.99	0.98				
	lysoPC a C26:1		0.75	0.43	0.44	0.0021	0.69	0.81	0.64	0.49	0.13	0.8				
	PC aa C34:3	12	0.87	0.23	0.16	0.0048	0.041	0.0001	0.77	0.0002	0.53	0.58	0.13	0.0001	0.25	0.0003
	PC aa C34:2		0.87	0.32	0.18	0.41	0.83	0.0001	0.35	0.0009	0.11	0.53				
	lysoPC a C18:1	13	0.84	0.63	0.44	0.0039	0.0003	0.4	0.065	0.02	0.52	0.31	0.52	0.15	0.0021	0.075
	lysoPC a C20:3		0.78	0.36	0.34	0.0002	0.062	0.0004	0.46	0.026	0.14	0.42				
	lysoPC a C20:4		0.86	0.29	0.20	0.0009	0.001	0.74	0.049	0.045	0.51	0.51				
	lysoPC a C16:0		0.91	0.76	0.36	0.66	0.001	0.32	0.32	0.15	0.25	0.29				
	PC ae C40:3	14	0.73	0.55	0.59	0.0001	0.033	0.0001	0.0001	0.0046	0.62	0.12	0.0001	0.0085	0.0001	0.27
	PC ae C36:1		0.75	0.53	0.52	0.0001	0.0001	0.0029	0.27	0.08	0.79	0.037				
	PC ae C34:2		0.69	0.34	0.47	0.0001	0.0001	0.57	0.27	0.082	0.84	0.049				
	PC aa C36:0		0.64	0.38	0.58	0.0001	0.0001	0.68	0.0062	0.18	0.18	0.59				
	PC ae C36:2		0.88	0.79	0.59	0.0001	0.0008	0.2	0.85	0.31	0.86	0.16				
	PC aa C40:2		0.74	0.51	0.53	0.0001	0.0001	0.0001	0.0047	0.56	0.42	0.28				
Sphingomyelins	SM C18:0	1	0.90	0.29	0.14	0.038	0.29	0.43	0.32	0.89	0.16	0.72	0.48	0.045	0.079	0.8
	SM (OH) C14:1		0.81	0.44	0.34	0.65	0.11	0.074	0.2	0.41	0.065	0.29				
	SM C20:2		0.81	0.38	0.32	0.0063	0.8	0.32	0.43	0.79	0.049	0.7				
	SM (OH) C24:1		0.77	0.29	0.32	0.081	0.095	0.004	0.2	0.13	0.21	0.68				
	SM C24:0		0.73	0.57	0.64	0.71	0.0002	0.047	0.022	0.64	0.28	0.3				
	SM (OH) C16:1		0.69	0.33	0.46	0.44	0.38	0.12	0.14	0.67	0.07	0.15				
	SM C16:0		0.65	0.57	0.81	0.0001	0.18	0.088	0.046	0.71	0.17	0.2				
	SM C26:0		0.58	0.31	0.61	0.0005	0.0001	0.0018	0.1	0.82	0.59	0.21				
	SM C24:1	2	0.88	0.21	0.15	0.0001	0.0001	0.0014	0.0003	0.11	0.62	0.097	0.0001	0.0057	0.0001	0.81
	SM C26:1		0.86	0.67	0.43	0.0001	0.0006	0.0098	0.023	0.95	0.2	0.35				
	SM (OH) C22:2		0.80	0.15	0.24	0.0001	0.0001	0.0008	0.0001	0.0033	0.2	0.01				
	SM (OH) C22:1		0.75	0.60	0.62	0.0001	0.033	0.059	0.27	0.76	0.084	0.36				
	SM C16:1	3	0.85	0.39	0.25	0.12	0.68	0.014	0.25	0.12	0.39	0.34	0.62	0.19	0.49	0.23
	SM C18:1		0.85	0.14	0.18	0.0007	0.093	0.96	0.67	0.37	0.5	0.72				
